# Prognostic impact of urokinase-type plasminogen activator system components in clear cell renal cell carcinoma patients without distant metastasis

**DOI:** 10.1186/1471-2407-14-974

**Published:** 2014-12-18

**Authors:** Susanne Fuessel, Kati Erdmann, Helge Taubert, Andrea Lohse-Fischer, Stefan Zastrow, Matthias Meinhardt, Karen Bluemke, Lorenz Hofbauer, Paolo Fornara, Bernd Wullich, Gustavo Baretton, Viktor Magdolen, Manfred P Wirth, Matthias Kotzsch

**Affiliations:** Department of Urology, Technische Universität Dresden, Fetscherstrasse 74, 01307 Dresden, Germany; Department of Urology, Universität Erlangen, Hartmannstrasse 14, 91054 Erlangen, Germany; Institute of Pathology, Technische Universität Dresden, Fetscherstrasse 74, 01307 Dresden, Germany; Institute of Legal Medicine, Martin Luther Universität Halle-Wittenberg, Franzosenweg 1, 06112 Halle, Germany; Department of Medicine III, Technische Universität Dresden, Fetscherstrasse 74, 01307 Dresden, Germany; Department of Urology, Martin Luther Universität Halle-Wittenberg, Ernst-Grube-Strasse 40, 06120 Halle, Germany; Clinical Research Unit, Department of Obstetrics and Gynecology, Technische Universität München, Ismaninger Strasse 22, 81675 München, Germany

**Keywords:** PAI-1, Prognostic biomarker, Renal cell carcinoma, uPA, uPAR, uPA system

## Abstract

**Background:**

Members of the urokinase-type plasminogen activator (uPA) system including uPA, its receptor uPAR and the plasminogen activator inhibitor 1 (PAI-1) play an important role in tumour invasion and progression in a variety of tumour types. Since the majority of clear cell renal cell carcinoma (ccRCC) shows distant metastasis at time of diagnosis or later, the interplay of uPA, uPAR and PAI-1 might be of importance in this process determining the patients’ outcome.

**Methods:**

Corresponding pairs of malignant and non-malignant renal tissue specimens were obtained from 112 ccRCC patients without distant metastasis who underwent tumour nephrectomy. Tissue extracts prepared from fresh-frozen tissue samples by detergent extraction were used for the determination of antigen levels of uPA, uPAR and PAI-1 by ELISA. Antigen levels were normalised to protein concentrations and expressed as ng per mg of total protein.

**Results:**

Antigen levels of uPA, uPAR, and PAI-1 correlated with each other in the malignant tissue specimens (r_s_=0.51-0.65; all *P*<0.001). Antigen levels of uPA system components were significantly higher in tissue extracts of non-organ confined tumours (pT3+4) compared to organ-confined tumours (pT1+2; all *P*<0.05). Significantly elevated levels of uPAR and PAI-1 were also observed in high grade ccRCC. When using median antigen levels as cut-off points, all three uPA system factors were significant predictors for disease-specific survival (DSS) in univariate Cox’s regression analyses. High levels of uPA and uPAR remained independent predictors for DSS with HR=2.86 (95% CI 1.07-7.67, *P*=0.037) and HR=4.70 (95% CI 1.51-14.6, *P*=0.008), respectively, in multivariate Cox’s regression analyses. A combination of high antigen levels of uPA and/or uPAR further improved the prediction of DSS in multivariate analysis (HR=14.5, 95% CI 1.88-111.1, *P*=0.010). Moreover, high uPA and/or uPAR levels defined a patient subgroup of high risk for tumour-related death in ccRCC patients with organ-confined disease (pT1+2) (HR=9.83, 95% CI 1.21-79.6, *P*=0.032).

**Conclusions:**

High levels of uPA and uPAR in tumour tissue extracts are associated with a significantly shorter DSS of ccRCC patients without distant metastases.

**Electronic supplementary material:**

The online version of this article (doi:10.1186/1471-2407-14-974) contains supplementary material, which is available to authorized users.

## Background

Renal cell carcinoma (RCC), comprising 3-4% of all malignant neoplasms, is the third most common urological tumour entity and the 13th most common malignancy worldwide [1, 2]. Clear cell RCC (ccRCC) is the most common and the most aggressive subtype of this disease [3]. About one third of the patients already present distant metastases at the time of diagnosis, whereas nearly another third develops metastases during the course of the disease [1, 4]. Although the application of target-directed therapeutics improved the outcome of patients with metastatic ccRCC their prognosis remains unfavourable [5].

Since the disease progression of RCC is still not sufficiently predictable by clinical and histopathological parameters, there is an urgent need for additional powerful biological prognostic factors that may help to refine individual risk stratification of RCC patients. A reliable prediction of outcome after nephrectomy by such markers would be valuable to tailor individualised follow-up schedules and to provide adjuvant therapies to patients at high risk of relapse [6]. Several clinicopathological parameters and molecular biomarkers or their combination have been described to be prognostically useful for RCC [7–9]. Nevertheless, further research and validation of these molecular markers for diagnostic and prognostic purposes in RCC patients are still necessary.

The urokinase-type plasminogen activator (uPA) system consisting mainly of uPA, the uPA receptor (uPAR) and the plasminogen activator inhibitor-1 (PAI-1) has been shown to play a key role in physiological and pathological pathways including carcinogenesis [10–12]. In addition to extracellular proteolysis, uPA in concert with uPAR and/or PAI-1 induces cell signaling pathways that affect multiple steps of cancer progression such as angiogenesis, cell growth, cell adhesion and migration, chemotaxis and cell survival [10, 11, 13, 14].

High expression levels of uPA and/or PAI-1 in tumour tissue extracts have been found to be strong predictors of poor prognosis in patients afflicted with different types of solid malignant tumours [15, 16]. High uPAR levels are also associated with poor prognosis in a variety of cancer types, however, the prognostic impact of uPAR expression is not as pronounced as that of uPA and PAI-1 [17, 18].

To date, only few studies exist that characterise the role of uPA system members in RCC and analyse their association with clinicopathological parameters and their impact on the outcome of RCC patients. High uPA, uPAR and/or PAI-1 protein expression in tumour tissue of RCC patients, detected by immunohistochemistry, was significantly associated with higher tumour stage, metastasis and poor disease-specific survival in a cohort of 106 RCC patients [19]. Furthermore, significant associations were observed between high immunoexpression of PAI-1 in tumour tissue of ccRCC patients and older age, advanced tumour stage, high nuclear grade, and disease progression [20, 21]. In survival analyses, strong PAI-1 immunostaining was associated with a shorter disease-free survival of ccRCC patients [20]. Furthermore, high PAI-1 immunoexpression was identified as an independent predictor of cancer-specific survival in a cohort of 172 ccRCC patients [21].

Increased uPA, uPAR and/or PAI-1 antigen levels in tumour tissue extracts, as determined by ELISA, were found to be related to high tumour grade of RCC [22, 23]. Hofmann et al. [24, 25], who also measured antigen levels of uPA, uPAR and PAI-1 in renal tissue extracts from RCC patients by ELISA, revealed all three factors as strong and independent predictors for early relapse.

Thus, the above-mentioned studies point to a rather strong clinical relevance of the members of the uPA-system in RCC, however, with some important limitations. Most of these data were obtained using tissue samples originating from different RCC subtypes, inconsistent cohort sizes, optimised cut-off points, the use of non-detergent tissue extracts or the three factors were not analysed in parallel. Therefore, in the present study, uPA, uPAR and PAI-1 antigen levels were determined in detergent tissue extracts derived from corresponding malignant and non-malignant renal tissues of 112 RCC patients of the clear-cell subtype without clinically detectable distant metastases at the time of diagnosis by ELISA. The antigen concentrations of uPA system components in tissue extracts were analysed for correlations between these factors themselves and for potential associations with clinicopathological parameters. In addition, we assessed the prognostic power of these markers for the prediction of disease-specific survival (DSS) and overall survival (OS) to validate their usefulness for outcome prediction of ccRCC patients without distant metastasis.

## Methods

### Study population

In total, 112 patients with ccRCC were included in this retrospective study that was approved by the institutional review boards of the Technische Universität Dresden and the Martin Luther University Halle-Wittenberg. Written informed consent was obtained from all patients. Patients were treated by partial or radical tumour nephrectomy between 1994 and 2001 at either the Department of Urology of the Technische Universität Dresden or the Department of Urology of the Martin Luther University Halle-Wittenberg. Tumours were restaged according to the UICC-classification 2010 [26]; tumour grading was performed according to Fuhrman et al. [27]. The patient cohort consisted of 65 men (58%) and 47 women (42%). Median age was 64.4 years (range 38–88 years). Organ-confined tumours (pT1+pT2) were found in 87 patients (78%), and non-organ confined tumours (pT3+pT4) in 25 patients (22%). 68 patients (61%) had low grade tumours (G1+G2), whereas 44 patients (39%) displayed high grade tumours (G3+G4). None of the patients included in the study showed clinically detectable distant metastasis at the time of diagnosis, but 6 patients (5%) had tumour-positive lymph nodes (pN1). Furthermore, patients with distant metastases detected within six months after primary surgery were excluded from the study.

Follow-up data were collected with regard to death of patients and cause of death. Median follow up for all patients comprised 101 months (range 9–188 months) with 38 deaths of any cause (33.9%; median survival 61.5 months) in the analysis of OS and with 21 patients (18.8%; median survival 32.0 months) who died of ccRCC in the analysis of DSS. All clinical and histopathological characteristics of the ccRCC patients are given in Table 1.

**Table 1:** **Clinical and histopathological characteristics of the ccRCC patients**

Clinicopathological parameters	No. patients (%)
**Total**	112
**Gender**	
male	65 (58.0)
female	47 (42.0)
**Age (years)**	
≤64	57 (50.9)
>64	55 (49.1)
**Tumour stage**	
pT1	73 (65.2)
pT2	14 (12.5)
pT3	22 (19.6)
pT4	3 (2.7)
**Tumour grade**	
G1	5 (4.5)
G2	63 (56.3)
G3	35 (31.2)
G4	9 (8.0)
**Lymph node status**	
positive	6 (5.4)
negative	106 (94.6)
**Disease-specific death (DSS)**	
alive	91 (81.2)
died of ccRCC	21 (18.8)
**Death of any cause (OS)**	
alive	74 (66.1)
died of any cause	38 (33.9)

### Determination of tissue antigen levels by ELISA

Matched pairs of malignant and non-malignant renal tissue specimens obtained directly after surgery were snap-frozen and stored in liquid nitrogen until further use. Detergent tissue extracts were prepared from frozen renal tissue specimens as previously described [28, 29]. The use of detergent-containing extraction buffer was recommended by the manufacturer, since it allows the reliable extraction of uPA, PAI-1 and the membrane-bound uPA receptor and the parallel determination of all three factors in one tissue extract [28, 30]. Briefly, after solubilisation of membrane-bound proteins using Tris-buffer containing the non-ionic detergent Triton X-100 (1%), cell debris was separated by centrifugation and the supernatant was stored at -80°C until use. The uPA, uPAR and PAI-1 antigen content in tissue extracts of ccRCC patients was determined applying commercially available ELISA kits (IMUBIND uPA ELISA # 894, IMUBIND uPAR ELISA # 893 and IMUBIND Tissue PAI-1 ELISA # 821; American Diagnostica/Sekisui Diagnostics, Stamford, CT, USA) according to the manufacturer’s instructions. IMUBIND ELISA tests for uPA and PAI-1 have previously been extensively validated in quality assessment trials by an EORTC study group [31]. The protein content of tumour tissue extracts was determined using the BCA protein assay (Sigma, Deisenhofen, Germany). Antigen concentrations in tissue extracts were expressed as ng analyte per mg of total protein.

### Statistical analyses

The distribution of protein levels of uPA, uPAR and PAI-1 in malignant and non-malignant tissue specimens is presented by boxplots. The Wilcoxon test was used to test for significant differences in antigen levels between both sample groups.

Correlations between continuous variables of the three uPA system components were assessed by Spearman's rank correlation coefficients (r_s_). The relationship of antigen levels with clinicopathological parameters was evaluated using the non-parametric Mann–Whitney test. DSS and OS of ccRCC patients were used as follow-up end points for survival analyses. The median levels were set as cut points to separate ccRCC patients in groups with low or high marker expression. For statistical analyses of the association between expression of uPA system components and patients’ prognosis univariate and multivariate Cox’s proportional hazard regression models were used to calculate the hazard ratio (HR) and its 95% confidence interval (CI) in the analysis of OS and DSS. The multivariate Cox’s regression models were adjusted to known clinical prognostic factors in ccRCC patients such as gender, age, tumour stage and tumour grade. Survival curves were generated using Kaplan-Meier analysis applying the log-rank test to look for differences in survival. All calculations were performed using the StatView 5.0 statistical package (SAS Institute, Cary, NC, USA). *P*-values < 0.05 were considered as statistically significant.

## Results

### Comparison of uPA system component levels in corresponding malignant and non-malignant tissue specimens of ccRCC patients

**Figure 1:**
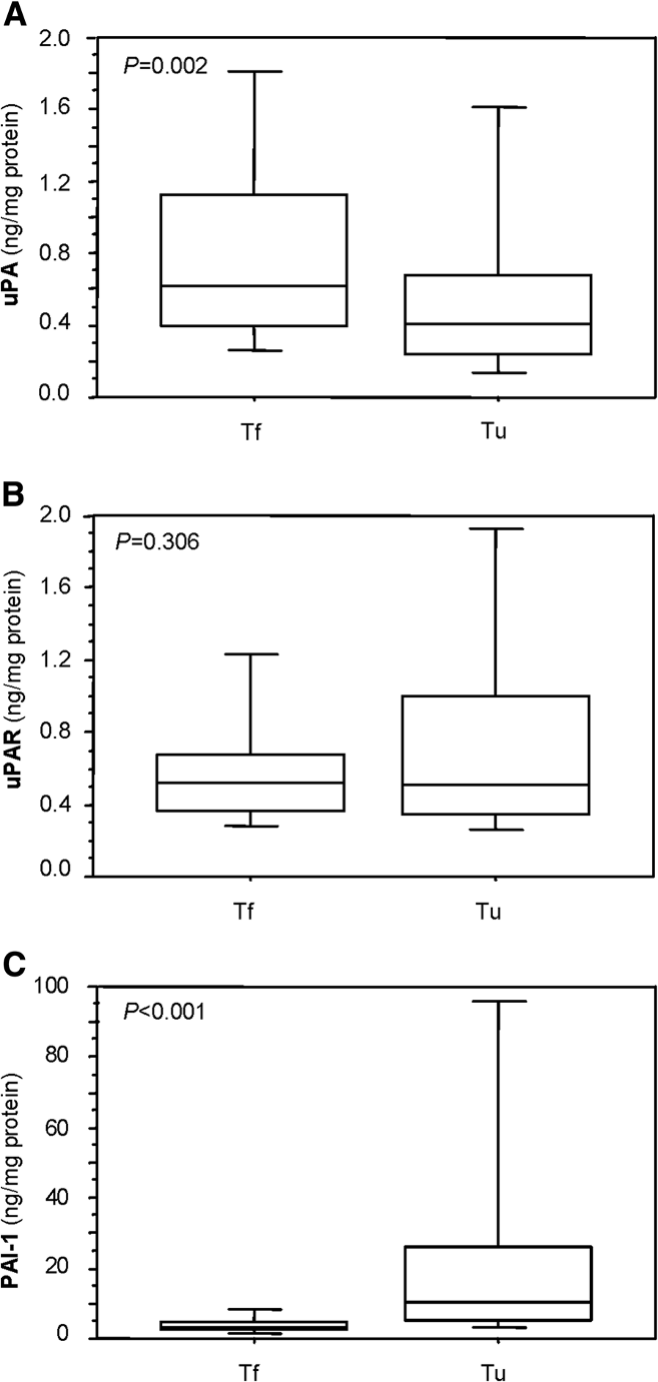
**Protein levels in matched pairs of malignant and non-malignant renal tissue specimens.** Distribution of protein levels of **A)** uPA, **B)** uPAR and **C)** PAI-1 in malignant (Tu) and non-malignant (Tf) renal tissues from 112 ccRCC patients assessed by ELISA is presented by boxplots. The boxes represent the 25th – 75th percentiles, the whiskers indicate the 10th and 90th percentiles. The median values are depicted as solid lines within the boxes. The Wilcoxon test was used to test for significant differences in antigen levels between Tu and Tf.

The median normalised antigen levels (ng/mg protein) of uPA, uPAR, and PAI-1 were 0.41 (range 0.03 - 7.44), 0.51 (range 0.16 - 8.31), and 10.3 (range 1.28 – 3738.3), respectively, in the ccRCC tissues. In tissue extracts of the adjacent non-malignant tissue median antigen levels of 0.61 (range 0.09 - 4.99), 0.52 (range 0.10 - 6.17), and 3.13 (range 1.17 – 34.5) were determined for uPA, uPAR, and PAI-1, respectively (Figure 1).

Significantly reduced antigen levels of uPA were observed in the tumour tissue specimens compared to the corresponding non-malignant tissues (*P* = 0.002), whereas PAI-1 antigen levels were significantly increased in tumour tissue specimens (*P* < 0.001). In contrast, no significant differences in protein levels between malignant and non-malignant tissue samples were observed for uPAR (*P* = 0.306). The distribution of the uPA, uPAR, and PAI-1 antigen levels in tissue extracts of the corresponding tissue pairs is shown in Figure 1.

Antigen levels of the uPA system components in tumour tissue specimens showed a moderate, significant correlation amongst each other. Spearman’s correlation coefficients of r_s_ = 0.51 (*P* < 0.001) were calculated for the association between uPA and uPAR, of r_s_ = 0.54 (*P* < 0.001) for uPA with PAI-1 and of r_s_ = 0.65 (*P* < 0.001) for uPAR with PAI-1. Conversely, only a low correlation (r_s_ < 0.28) was observed between uPA system component levels in non-malignant tissue specimens.

### Association of uPA system component levels in tumour tissue with clinicopathological parameters of ccRCC patients

Associations of uPA, uPAR and PAI-1 antigen levels in tumour tissue extracts with relevant clinicopathological factors are summarised in Table 2. Levels of uPA in tumour tissue samples differed significantly between male and female patients (*P* = 0.002), whereas the association of uPAR and PAI-1 with gender was not significant. Significantly higher levels of uPAR, but not of uPA and PAI-1, were observed in patients at higher age (older than the median of 64 years) compared to younger patients (*P* = 0.029). Furthermore, antigen levels of all three uPA system factors were significantly elevated in tumour tissue from non-organ confined tumours (pT3+4) compared to organ-confined tumours (pT1+2; all *P* < 0.05). High grade tumours (G3+4) displayed significantly increased levels of uPAR (*P* = 0.008) and PAI-1 (*P* = 0.011) in comparison to low grade tumours (G1+2). However, uPA levels in tumour tissue extracts were not related to tumour grade (Table 2).

**Table 2:** **Protein levels of uPA system components in tumour tissue specimens in relation to clinicopathological parameters of the ccRCC patients**

Clinicopathological parameters	No. patients	uPA ^a^	uPAR ^a^	PAI-1 ^a^
**Total**	112	0.41 (0.4)	0.51 (0.7)	10.31 (20.7)
**Gender** ^**b**^		*P* = 0.002	*P* = 0.065	*P* = 0.056
male	65	0.47 (0.6)	0.60 (0.7)	12.58 (29.1)
female	47	0.30 (0.3)	0.42 (0.5)	7.18 (13.9)
**Age (years)** ^**b**^		*P* = 0.117	*P* = 0.029	*P* = 0.242
≤64	57	0.36 (0.3)	0.43 (0.4)	8.98 (13.4)
>64	55	0.46 (0.7)	0.63 (0.7)	11.74 (54.7)
**Tumour stage** ^**b**^		*P* = 0.003	*P* = 0.021	*P* = 0.010
pT1+2	87	0.36 (0.3)	0.47 (0.5)	8.33 (14.0)
pT3+4	25	0.63 (0.9)	0.81 (1.4)	18.05 (62.3)
**Tumour grade** ^**b**^		*P* = 0.122	*P* = 0.008	*P* = 0.011
G1+2	68	0.40 (0.3)	0.43 (0.5)	7.83 (12.8)
G3+4	44	0.43 (0.9)	0.68 (1.1)	14.57 (62.9)
**Disease-specific survival** ^**b**^		*P* = 0.220	*P* = 0.002	*P* = 0.007
alive	91	0.37 (0.4)	0.46 (0.5)	7.91 (14.4)
died of ccRCC	21	0.50 (0.7)	0.94 (0.8)	16.08 (65.0)
**Overall survival** ^**b**^		*P* = 0.568	*P* = 0.013	*P* = 0.037
alive	74	0.38 (0.4)	0.45 (0.4)	7.59 (17.7)
died of any cause	38	0.46 (0.5)	0.69 (0.7)	12.57 (57.1)

^a^Median values (interquartile range), ng analyte/mg protein; ^b^Mann-Whitney test.

### Association of uPA system component levels with survival of ccRCC patients

**Figure 2:**
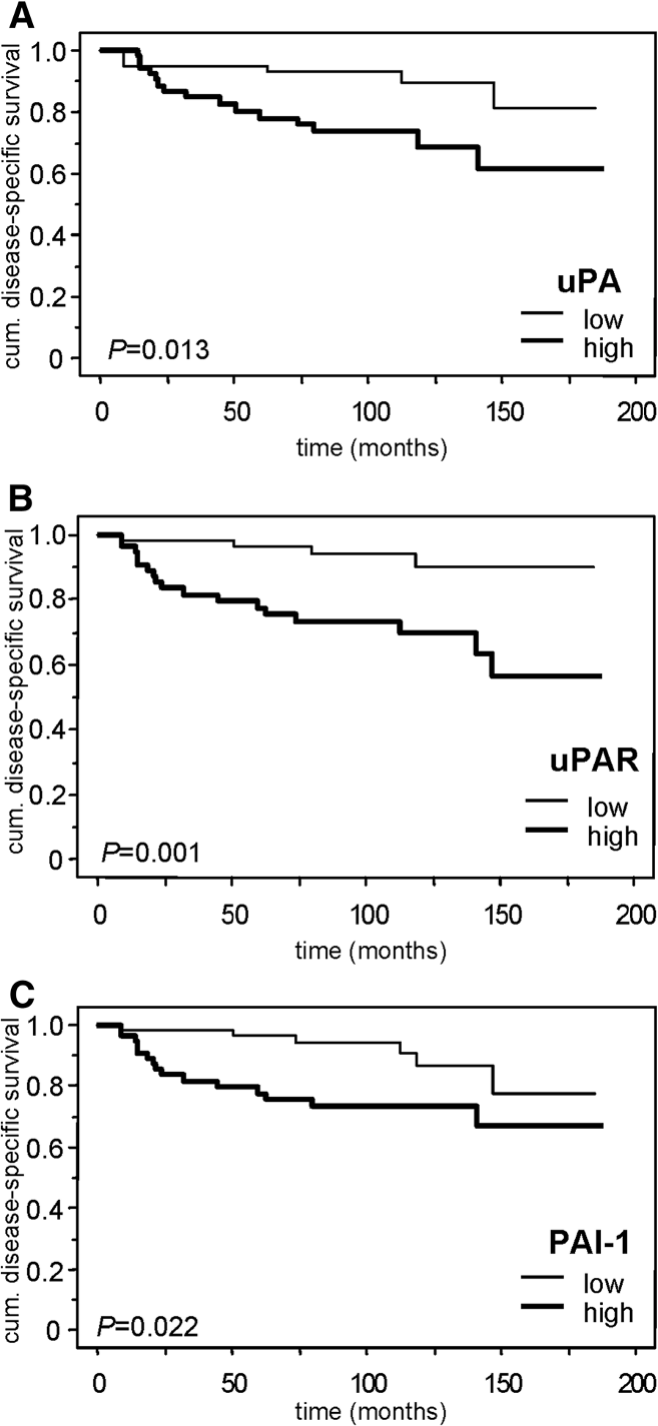
**Disease-specific survival of the ccRCC patients in relation to the uPA system components.** Kaplan-Meier curves show the dependence of disease-specific survival (DSS) on the protein levels of **A)** uPA, **B)** uPAR and **C)** PAI-1. Differences in DSS between patients with low and high levels of the uPA system components were assessed using the log-rank test.

For statistical analyses of the impact of uPA system components on patients’ survival, the median protein expression levels of uPA, uPAR, and PAI-1 were used as cut-off points to classify the ccRCC patients into groups with low or high antigen levels in tumour tissue extracts. In univariate Cox’s regression analyses, high antigen levels of all three uPA system components in tumour tissue were significantly associated with both shorter DSS and OS of ccRCC patients, except for uPA, for which an association of high antigen levels was observed with OS only (Additional file 1: Table S1). These findings were confirmed by the log-rank test which also revealed significantly shorter DSS of patients with high levels of uPA system components (*P* = 0.013, *P* = 0.001, and *P* = 0.022 for uPA, uPAR, and PAI-1, respectively) as exemplarily visualised by the respective survival curves (Figure 2).

Furthermore, the relevant clinicopathological parameters age, tumour stage and tumour grade were univariate predictors for DSS. However, in univariate analysis of OS only age and tumour grade reached statistical significance, whereas gender did not affect outcome of ccRCC patients in neither DSS nor OS (Additional file 1: Table S1).

The independent relationship of uPA system components with DSS and OS of ccRCC patients was evaluated by multivariate Cox’s regression analyses by adding these factors separately to a base model consisting of clinicopathological parameters including gender, age, tumour stage and tumour grade. Strikingly, uPA and uPAR antigen levels in tumour tissue extracts were significantly associated with shorter DSS, whereas none of the clinicopathological parameters showed a significant predictive value towards DSS (Table 3). For ccRCC patients with either high uPA (HR = 2.86; 95% CI 1.07-7.67; *P* = 0.037) or uPAR antigen levels (HR = 4.70; 95% CI 1.51-14.6; *P* = 0.008) we observed a significantly increased risk of cancer-related death compared with those patients who displayed low uPA or uPAR antigen levels in ccRCC tissue (Table 3). PAI-1 antigen levels were associated with DSS only by trend in multivariate analysis (HR = 2.59; 95% CI 0.95-7.08; *P* = 0.064). When adding all three factors simultaneously to the basic model, only uPAR remained an independent prognostic factor for DSS with HR = 3.54 (95% CI 1.02-12.2, *P* = 0.046). In contrast, uPA system members did not emerge as independent prognosticators for OS (Table 3). Among the clinicopathological parameters only age displayed an independent prognostic value for OS (Table 3).

**Table 3:** **Associations of uPA system components in tumour tissue specimens with disease-specific survival (DSS) and overall survival (OS) of patients with ccRCC (n = 112) assessed by multivariate Cox’s regression analysis**

Factor	No. cases	Disease-specific survival HR (95% CI) ^a^	***P***	Overall survival HR (95% CI) ^a^	***P***
**Gender**					
male	65	1		1	
female	47	0.93 (0.38-2.28)	0.880	1.65 (0.79-3.45)	0.179
**Age (years)**					
≤64	57	1		1	
>64	55	2.31 (0.89-5.99)	0.085	2.52 (1.26-5.07)	0.009
**Tumour stage**					
pT1+2	87	1		1	
pT3+4	25	1.85 (0.73-4.69)	0.192	1.17 (0.56-2.44)	0.670
**Tumour grade**					
G1+2	68	1		1	
G3+4	44	2.08 (0.83-5.19)	0.118	1.61 (0.82-3.14)	0.165
**uPA** ^**b**^					
low	53	1		1	
high	59	2.86 (1.07-7.67)	0.037	1.28 (0.66-2.51)	0.467
**uPAR** ^**b**^					
low	55	1		1	
high	57	4.70 (1.51-14.6)	0.008	1.60 (0.80-3.23)	0.185
**PAI-1** ^**b**^					
low	56	1		1	
high	56	2.59 (0.95-7.08)	0.064	1.71 (0.85-3.46)	0.135

^a^HR: hazard ratio; 95% CI: 95% confidence interval of multivariate Cox’s regression analysis; uPA system factors were separately added to the base model consisting of gender, age, tumour stage and tumour grade.

^b^Dichotomised into groups with high and low levels of uPA system factors by the median values.

In addition, we performed statistical analyses in the subgroup of ccRCC patients with organ-confined tumours (pT1+2; n = 87). Here, the clinicopathological variables were not significantly related to the patients’ survival, except for age, which was a significant predictor for OS in uni- and multivariate analyses (Additional file 1: Tables S2 and S3). Likewise, no significant associations were observed between uPA system component levels in tumour tissue extracts and OS (Additional file 1: Tables S2 and S3). On the contrary, in the analysis of DSS we found an association between high uPAR antigen levels and an increased risk of cancer-related death with a trend towards significance for patients with organ-confined tumours in either univariate or multivariate Cox regression analysis with HRs of 3.15 (95% CI 0.97-10.2, *P* = 0.057) and 3.09 (95% CI 0.88-10.8, *P* = 0.077), respectively (Additional file 1: Tables S2 and S3).

### Analysis of combined uPA system component levels in tumour tissues for survival of ccRCC patients

In the next step, we assessed whether the pair-wise combination of uPA, uPAR and PAI-1 antigen levels could improve the prognostic power of either single marker. For this, the ccRCC patient cohort was divided into two groups: a group with low levels of each of both markers analysed, and another with high levels of one or both markers. In the whole patient cohort, the marker combinations of high antigen levels of either uPA and/or uPAR, uPA and/or PAI-1 or uPAR and/or PAI-1 were found to be significantly associated with shorter DSS in univariate Cox’s regression analyses (Additional file 1: Table S1). Particularly, the co-detection of high antigen levels of uPA and/or uPAR added significant prognostic information for DSS in ccRCC patients with a HR of 12.6 (95% CI 1.69-94.1; *P* = 0.013). Moreover, in multivariate analyses using the base model mentioned above (comprising gender, age, tumour stage and tumour grade), we found that combined high antigen levels of uPA and/or uPAR resulted in an even better prediction of DSS represented by a HR of 14.5 (95% CI 1.88-111.1, *P* = 0.010), which was independent of other factors (Table 4). The combined antigen levels of high uPA and/or PAI-1 (HR = 4.24, 95% CI 1.21-14.9, *P* = 0.024) did also provide additional prognostic information (Table 4).

**Table 4:** **Associations of combinations of uPA system components in tumour tissue specimens with disease-specific survival (DSS) and overall survival (OS) of patients with ccRCC (n = 112) assessed by multivariate Cox’s regression analysis**

Factor	No. cases	Disease-specific survival HR (95% CI) ^a^	***P***	Overall survival HR (95% CI) ^a^	***P***
**uPA/uPAR** ^**b**^					
uPA and uPAR low	41	1		1	
uPA and/or uPAR high	71	14.5 (1.88-111.1)	0.010	1.34 (0.64-2.82)	0.438
**uPA/PAI-1** ^**b**^					
uPA and PAI-1 low	43	1		1	
uPA and/or PAI-1 high	69	4.24 (1.21-14.9)	0.024	1.63 (0.78-3.40)	0.196
**uPAR/PAI-1** ^**b**^					
uPAR and PAI-1 low	40	1		1	
uPAR and/or PAI-1 high	72	3.47 (0.98-12.2)	0.053	1.56 (0.73-3.36)	0.255

^a^HR: hazard ratio; 95% CI: 95% confidence interval of multivariate Cox’s regression analysis; combinations of uPA system factors were separately added to the base model consisting of gender, age, tumour stage and tumour grade.

^b^Dichotomised into groups with high and low levels of uPA system factors by the median values.

In addition, in the subgroup of patients with organ-confined disease, those with low antigen levels of both uPA and uPAR were characterised by a longer DSS than patients with tumours with high levels of one or both markers (Table 5). Patients with pT1+2 tumours with high levels of uPA and/or uPAR showed a significantly increased risk of cancer-related death (HR = 9.83; 95% CI 1.21-79.6, *P* = 0.032) compared to pT1+2 patients who had low uPA and uPAR levels (Table 5). Thus, a pronounced additive effect on prognosis of pT1+2 ccRCC patients, i.e. patients with an assumed lower risk of cancer-related death, was identified when combining uPA and uPAR antigen levels in tumour tissue. The other combinations of high uPA and/or PAI-1 or uPAR and/or PAI-1 did not provide any additional prognostic information for ccRCC patients with organ-confined disease (Table 5).

**Table 5:** **Association of the combination of uPA system component levels in tumour tissue specimens with disease-specific survival** (**DSS) and overall survival** (**OS) in the subgroup of ccRCC patients with organ-confined tumours** (**tumour stage pT1+2, n = 87) assessed by multivariate Cox’s regression analysis**

Factor	No. cases	Disease-specific survival HR (95% CI) ^a^	***P***	Overall survival HR (95% CI) ^a^	***P***
**uPA/uPAR** ^**b**^					
uPA and uPAR low	35	1		1	
uPA and/or uPAR high	52	9.83 (1.21-79.6)	0.032	1.03 (0.45-2.37)	0.942
**uPA/PAI-1** ^**b**^					
uPA and PAI-1 low	37	1		1	
uPA and/or PAI-1 high	50	3.23 (0.79-13.1)	0.101	1.30 (0.56-3.02)	0.541
**uPAR/PAI-1** ^**b**^					
uPAR and PAI-1 low	35	1		1	
uPAR and/or PAI-1 high	52	2.68 (0.67-10.8)	0.164	1.39 (0.58-3.29)	0.460

^a^HR: hazard ratio; 95% CI: 95% confidence interval of multivariate Cox’s regression analysis; combinations of uPA system factors were separately added to the base model consisting of gender, age and tumour grade.

^b^Dichotomised into groups with high and low levels of uPA system factors by the medians.

For overall survival, none of the marker combinations represented a significant predictor in univariate or multivariate Cox’s regression analyses neither in the whole cohort nor in the subgroup of ccRCC patients with organ-confined disease (Additional file 1: Tables S1 and S2, Tables 4 and 5).

## Discussion

In the present study, we aimed at analysing the protein levels of uPA system components in tissue extracts from matched malignant and corresponding non-malignant tissue kidney specimens in a cohort of 112 ccRCC patients without distant metastasis and at assessing their potential associations with clinicopathological parameters and the prognostic relevance in ccRCC patients.

The antigen levels of uPA, uPAR and PAI-1 (ng analyte/mg of total protein) determined in detergent extracts of corresponding malignant and non-malignant kidney tissues in our study were comparable to those reported in previous studies applying the same commercially available ELISA kits (IMUBIND from Sekisui/American Diagnostica) or using in-house ELISA formats (see Table 6). Conversely, in cytosolic tumour tissue extracts, considerably lower uPA concentrations were measured, whereas the PAI-1 antigen levels were comparable [22].

**Table 6:** **Overview of median protein levels of uPA, uPAR and PAI-1 determined by different ELISA formats in malignant and non-malignant renal tissue specimens**

Parameter	Hofmann et al. [24], [25]	Swiercz et al. [23] ^a^	Chautard et al. [22] ^b,c^	Span et al. [32]	Present study
**Number and type of Tu tissue samples**	152 RCC (not specified): 105 for uPA, 49 for uPAR, 96 for PAI-1	52 RCC (not specified)	100 RCC: 85 ccRCC, 11 papillary RCC, 4 sarcomatoid	55 RCC (not specified)	112 ccRCC
**Number and type of Tf tissue samples**	matched normal tissues from RCC kidneys	n = 28: 3 RCC-free kidneys, 18 normal tissues from RCC-kidneys, 7 tissues adjacent to RCC	matched normal tissues from RCC kidneys	matched normal tissues from RCC kidneys	matched normal tissues from RCC kidneys
**ELISA for uPA**	American Diagnostica	American Diagnostica	BYK Sangtec Medical (Nichols Laboratory)	self-designed ELISA	American Diagnostica
**ELISA for uPAR**	American Diagnostica	American Diagnostica	n.d.	n.d.	American Diagnostica
**ELISA for PAI-1**	self-designed ELISA	American Diagnostica	American Diagnostica,	self-designed ELISA	American Diagnostica
**Tissue processing**	detergent tissue extracts	detergent tissue extracts	cytosolic tissue extracts	detergent tissue extracts	detergent tissue extracts
**uPA**					
in Tu	0.29	n.p.	0.091 (0.043 in ccRCC)	0.50	0.41
in Tf	0.286	n.p.	0.078	0.71	0.61
Difference between Tu *vs* Tf	n.s. (Tu = Tf)	*P* = 0.01 (Tu > Tf)	n.s. (Tu > Tf)	n.s. (Tu < Tf)	*P* = 0.001 (Tu < Tf)
**uPAR**					
in Tu	0.78	n.p.	n.d.	n.d.	0.51
in Tf	0.30	n.p.	n.d.	n.d.	0.52
Difference between Tu *vs* Tf	*P* = 0.018 (Tu > Tf)	*P* = 0.007 (Tu > Tf)	n.d.	n.d.	n.s. (Tu = Tf)
**PAI-1**					
in Tu	11.08	n.p.	40.8 (38.7 in ccRCC)	7.26	10.3
in Tf	4.221	n.p.	5.8	0.92	3.13
Difference between Tu *vs* Tf	*P* = 0.02 (Tu > Tf)	*P* = 0.001 (Tu > Tf)	*P* < 0.001 (Tu > Tf)	*P* < 0.001 (Tu > Tf)	*P* = 0.001 (Tu > Tf)

n.d.: not determined; n.p.: not provided; n.s.: not significant; Tf: non-malignant tissues; Tu: malignant tissues.

^a^Only *P*-values were given for differences between malignant and non-malignant tissue samples, which are presented in box plots.

^b^Results are converted from pg/mg to ng/mg.

^c^Mean levels are shown since medians were not provided.

Levels of uPA, uPAR and PAI-1 were normalised to total protein concentration and expressed as ng analyte per mg of total protein.

The concentration of uPA system components has been reported to differ between malignant and non-malignant renal tissues (see Table 6). All mentioned studies including our present analyses demonstrated significantly higher protein levels of PAI-1 in RCC tissues compared to normal renal tissues (see Table 6). With regard to uPA and uPAR, the observations in the different studies vary. While Swiercz et al. [23] observed significantly elevated uPA levels in the tumour specimens, others found no significant differences between malignant and non-malignant renal tissues [22, 24, 25, 32]. In contrast, in our study uPA protein levels were significantly decreased in ccRCC tissues compared to the corresponding non-malignant specimens. The reasons for these discrepancies between the different reports remain unclear so far. Hofmann et al. [24, 25], who used the same strategy and analysed a similar number of patients as we did, measured comparable uPA levels in the corresponding renal tissue specimens, whereas Span et al. [32] obtained a similar trend of uPA levels in malignant and non-malignant renal tissues as in our study, which did not reach statistical significance (*P* = 0.081).

For uPAR, we found no differences between corresponding malignant and non-malignant tissue of ccRCC patients. In contrast, significantly higher uPAR antigen concentrations have been observed in RCC tissues compared to benign renal tissue in previous reports [23, 24]. These discrepancies might originate from differences in the numbers of analysed tissue samples. While 112 pairs of corresponding malignant and non-malignant renal tissues were used in our study, Hofmann et al. [24] assessed uPAR levels only in 49 matched tissue pairs and Swiercz et al. [23] compared 52 RCC tissues with 28 normal tissues of different origin (Table 6). Furthermore, levels of uPA system components might differ between the RCC subtypes. We analysed only patients with ccRCC, but no information on the subtype is given in the mentioned reports [23, 24]. Interestingly, Chautard et al. [22] observed clear differences in uPA and PAI-1 levels between tissue specimens from ccRCC (n = 85), papillary RCC (n = 11) and sarcomatoid RCC (n = 4) indicating that the RCC subtype is of importance and the use of mixed patient cohorts hampers the drawing of definite conclusions (Table 6).

In the tumour tissues, we found significant, moderately high correlations between the antigen levels of all three components of the uPA system (r_s_ values between 0.51 and 0.65). Similar positive correlations have in fact been previously reported between uPA, uPAR, and PAI-1 in tumour tissue extracts of RCC patients [24, 25]. Strikingly, significant correlations between antigen levels of these factors in tumour tissues have been also observed in various other types of cancer [33–35]. The regulated expression of uPA system components in a concerted manner in tumour growth and metastasis may be attributed to the various interactions within the uPA system [11, 16]. In contrast, we found only low correlations between uPA system components in non-malignant renal tissues, which is in line with observations on tumour-free samples of other tumour entities [32, 36]. This may be caused by various mechanisms of expression regulation and functional interplay between these factors under physiological conditions [11].

Only few studies have analysed the protein levels of uPA system components in RCC specimens with regard to their dependence on clinicopathological parameters and to their impact on prognosis of RCC patients. In the present study, we observed significantly elevated antigen levels of all three uPA system components in tissue specimens from non-organ confined tumours compared to organ-confined tumours. Furthermore, significantly increased levels of uPAR and PAI-1 could be detected in high grade tumours in comparison to low grade tumours. In accordance to our results, Swiercz et al. [23] reported elevated protein levels of uPA, uPAR and PAI-1 in tissue extracts from high grade RCC. Chautard et al. [22] also detected a gradual increase of uPA and PAI-1 protein levels in RCC extracts with higher tumour grade and stage, whereas only the relation between PAI-1 and grade reached statistical significance. A positive relation between the immunoreactive scores of uPA, uPAR or PAI-1 and tumour grade, tumour stage or metastasis was described by Ohba et al. [19] who performed immunohistochemical analyses on 106 RCC patients. Two other immunohistochemical studies on ccRCC patients also described significant positive associations of PAI-1 staining with tumour grade as well as with tumour stage [20, 21]. On the contrary, the studies by Hofmann et al. [24, 25] did not reveal any association between protein levels of uPA, uPAR or PAI-1 in tissue extracts and tumour grade or stage.

Moreover, data on the influence of the protein expression of uPA system members on survival of RCC patients are partially contradictory depending on the detection method used [19, 22, 24]. In the present study, all three uPA system components were significant predictors for DSS in univariate Cox’s regression analyses and high levels of uPA and uPAR remained independent prognostic factors for DSS in multivariate analyses. The combination of both factors further improved the prediction of DSS in multivariate analysis considerably (Table 4; HR=14.5, 95% CI 1.88-111.1, *P*=0.010). These results are in line with the data of the immunohistochemical analyses reported by Ohba et al. [19] who revealed that increased protein levels of all three factors were associated with poor DSS. Nevertheless, only PAI-1 emerged as independent predictive factor in their multivariate Cox’s regression analyses [19].

In the report of Hofmann et al. [24] who considered – in contrast to our study – the time from surgery to tumour progression, all three factors appeared also as strong predictors for a higher risk of relapse. In contrast, uPA and PAI-1 protein levels in cytosolic tissue extracts were not independently predictive for metastasis as reported by Chautard et al. [22] but elevated uPA levels were significantly associated with a shorter disease-free survival (DFS) in univariate Cox’s regression analyses. Similarly, Choi et al. [20] described a significant relation between high PAI-1 immunoexpression and a shorter DFS, albeit this could not be confirmed in multivariate Cox’s regression analyses. In comparison to this, the immunohistochemical study by Zubac et al. [21] revealed PAI-1 as a significant prognostic factor for disease-specific survival both in univariate and multivariate Cox’s regression analyses. Taken together, previous studies demonstrated a prognostic potential of uPA and uPAR, and most obviously, for PAI-1 protein levels in RCC tissues. In contrast, high levels of uPA and uPAR in ccRCC extracts were shown to be associated with a significantly shorter DSS of ccRCC patients in our study. The differing study results may originate from variances in the applied methods and used cut-off points as well as in the patient cohorts with regard to the inclusion of patients with diverse RCC subtypes, metastatic status and follow-up endpoints.

Finally, we evaluated the predictive value of the three uPA system components for patients with an organ-confined tumour who are usually expected to have a favourable outcome but still can progress after tumour nephrectomy given the highly aggressive nature of ccRCC. Surprisingly, the DSS of those patients could be further stratified on the basis of combined tissue protein levels of uPA and uPAR (Additional file 1: Table S2, Table 5). The HR of 9.83 (*P* = 0.032) obtained for patients with high levels of uPA and/or uPAR in multivariate analysis in the pT1+2 subgroup further emphasises the potential prognostic utility of uPA and uPAR protein expression for patients with ccRCC. Based on these findings, it might be also of interest to specifically determine the concentration of the uPA/uPAR complex with hybrid sandwich-type ELISAs and its relation to prognosis of the patients [37].

Moreover, Zubac et al. [21] could show that PAI-1 immunoexpression turned out to be a highly significant predictor for metastatic relapse in patients with organ-confined RCC. These results suggests that the determination of protein levels of uPA system components in patients with pT1 and pT2 tumours may provide additional prognostic information that may allow for individual, risk-adapted therapy decisions. Patients at higher risk of relapse could be treated by targeted agents in an adjuvant setting as currently evaluated in a number of ongoing prospective clinical trials [6]. For primary breast cancer, the determination of uPA and PAI-1 antigen levels in tumour tissue extracts has already entered clinical practice for risk stratification and individual therapy decisions in patients with lymph node-negative disease [16, 38]. This approach might be also imaginable for ccRCC patients without distant metastasis, particularly for those with organ-confined tumours, but needs further elucidation in prospective clinical trials.

## Conclusions

The present study, to our knowledge, is the first report on the parallel assessment of protein levels of uPA, uPAR and PAI-1 in detergent tissue extracts of renal tissue specimens in a consistent cohort of 112 patients with ccRCC and without clinically detectable distant metastasis at the time of diagnosis. Comparable previous studies were performed using tissue samples originating from different RCC subtypes and follow-up endpoints, inconsistent cohort sizes, optimised cut-off points or non-detergent tissue extracts or analysed not all three factors in parallel (see Table 6). Our study tried to overcome these shortcomings, and revealed a considerable, independent prognostic potential of uPA and uPAR protein levels in tumour tissue extracts for DSS. Best prediction of DSS might be achieved by the combination of elevated protein levels of uPA and/or uPAR compared to low levels of both factors. Particularly, this holds true for the subgroup of ccRCC patients with organ-confined tumours, whose outcome in general is assumed to be favourable but can be further stratified on the basis of the protein levels of uPA and uPAR. Despite the strengths of our retrospective study on 112 ccRCC patients, future prospective validation studies have to elucidate independently the prognostic value of the uPA system components for ccRCC patients.

## Electronic supplementary material

Additional file 1: Table S1: Univariate Cox’s regression analysis for disease-specific survival (DSS) and overall survival (OS) in patients with ccRCC (n = 112). Table S2: Univariate Cox’s regression analysis for disease-specific survival (DSS) and overall survival (OS) in the subgroup of ccRCC patients with organ-confined tumours (tumour stage pT1+2, n = 87). Table S3: Association of uPA system component levels in tumour tissue with disease-specific survival (DSS) and overall survival (OS) in the subgroup of ccRCC patients with organ-confined tumours (tumour stage pT1+2, n = 87) assessed by multivariate Cox’s regression analysis. (PDF 184 KB)

## Abbreviations

ccRCC: Clear cell renal cell carcinoma
CI: Confidence interval
DSS: Disease-specific survival
ELISA: Enzyme-linked immunosorbent assay
G: Grade
HR: Hazard ratio
OS: Overall survival
PAI-1: Plasminogen activator inhibitor 1
pN: Pathological lymph node status
pT: Pathological tumour stage
r_s_: Spearman’s correlation coefficient
UICC: Union for International Cancer Control
uPA: Urokinase-type plasminogen activator
uPAR: uPA receptor.
