# Supplementary material for: Prognostic impact of urokinase-type plasminogen activator system components in clear cell renal cell carcinoma patients without distant metastasis
Source: BMC Cancer. 2014 Dec 18;14:974. doi: 10.1186/1471-2407-14-974 (PMC4301943; doi:10.1186/1471-2407-14-974)
Supplement: Supplementary file 1 — Additional file 1: Table S1: Univariate Cox’s regression analysis for disease-specific survival (DSS) and overall survival (OS) in patients with ccRCC (n = 112). Table S2: Univariate Cox’s regression analysis for disease-specific survival (DSS) and overall survival (OS) in the subgroup of ccRCC patients with organ-confined tumours (tumour stage pT1+2, n = 87). Table S3: Association of uPA system component levels in tumour tissue with disease-specific survival (DSS) and overall survival (OS) in the subgroup of ccRCC patients with organ-confined tumours (tumour stage pT1+2, n = 87) assessed by multivariate Cox’s regression analysis. (PDF 184 KB) [file 12885_2014_5150_MOESM1_ESM.pdf]

Additional file 1: Table S1: Univariate Cox's regression analysis for disease-specific survival (DSS) and overall survival (OS) of patients with ccRCC (n = 112).

Factor	No. cases	Disease-specific survival HR (95% CI)[a]	*P*	Overall survival HR (95% CI)[a]	*P*
**Gender**					
male	65	1		1	
female	47	1.10 (0.45-2.65)	0.839	1.81 (0.88-3.72)	0.109
**Age (years)**					
≤ 64	57	1		1	
> 64	55	2.85 (1.13-7.21)	0.027	2.82 (1.43-5.56)	0.003
**Tumour stage**					
pT1+2	87	1		1	
pT3+4	25	2.50 (1.03-6.06)	0.042	1.68 (0.83-3.38)	0.150
**Tumour grade**					
G1+2	68	1		1	
G3+4	44	2.65 (1.10-6.40)	0.030	2.01 (1.06-3.81)	0.033
**uPA**[b]					
low	53	1		1	
high	59	3.11 (1.21-8.04)	0.019	1.61 (0.85-3.05)	0.148
**uPAR**[b]					
low	55	1		1	
high	57	5.03 (1.69-15.0)	0.004	2.03 (1.05-3.93)	0.036
**PAI-1**[b]					
low	56	1		1	
high	56	2.88 (1.12-7.44)	0.029	1.96 (1.01-3.79)	0.046
**uPA/uPAR**[b]					
uPA and uPAR low	41	1		1	
uPA and/or uPAR high	71	12.6 (1.69-94.1)	0.013	1.58 (0.78-3.19)	0.202
**uPA/PAI-1**[b]					
uPA and PAI-1 low	43	1		1	
uPA and/or PAI-1 high	69	4.42 (1.80-11.1)	0.017	1.88 (0.93-3.79)	0.080
**uPAR/PAI-1**[b]					
uPAR and PAI-1 low	40	1		1	
uPAR and/or PAI-1 high	72	3.80 (1.12-12.9)	0.032	1.79 (0.87-3.69)	0.114

[a]HR: hazard ratio; 95% CI: 95% confidence interval of univariate Cox's regression analysis

[b]Dichotomised into groups with high and low levels of uPA system factors in tumour tissue by the median values.

Additional file 1: Table S2: Univariate Cox's regression analysis for disease-specific survival (DSS) and overall survival (OS) in the subgroup of ccRCC patients with organ-confined tumours (tumour stage pT1+2, n = 87).

Factor	No. cases	Disease-specific survival HR (95% CI) [a]	*P*	Overall survival HR (95% CI) [a]	*P*
**Gender**					
male	47	1		1	
female	40	0.92 (0.31-2.73)	0.874	1.87 (0.82-4.28)	0.137
**Age (years)**					
≤ 64	48	1		1	
> 64	39	2.91 (0.92-9.18)	0.068	2.83 (1.29-6.21)	0.009
**Tumour grade**					
G1+2	58	1		1	
G3+4	29	1.68 (0.56-5.00)	0.352	1.56 (0.73-3.33)	0.254
**uPA[b]**					
low	51	1		1	
high	36	1.86 (0.62-5.55)	0.266	1.15 (0.53-2.49)	0.717
**uPAR[b]**					
low	49	1		1	
high	38	3.15 (0.97-10.2)	0.057	1.52 (0.71-3.24)	0.277
**PAI-1[b]**					
low	48	1		1	
high	39	1.60 (0.54-4.77)	0.398	1.49 (0.70-3.18)	0.299
**uPA/uPAR[b]**					
uPA and uPAR low	35	1		1	
uPA and/or uPAR high	52	8.80 (1.14-67.8)	0.037	1.30 (0.59-2.84)	0.513
**uPA/PAI-1[b]**					
uPA and PAI-1 low	37	1		1	
uPA and/or PAI-1 high	50	2.90 (0.80-10.6)	0.106	1.54 (0.70-3.37)	0.278
**uPAR/PAI-1[b]**					
uPAR and PAI-1 low	35	1		1	
uPAR and/or PAI-1 high	52	2.53 (0.70-9.19)	0.159	1.55 (0.70-3.45)	0.284

[a]HR: hazard ratio; 95% CI: 95% confidence interval of univariate Cox's regression analysis

[b]Dichotomised into groups with high and low levels of uPA system factors in tumour tissue by the median values.

Additional file 1: Table S3: Association of uPA system component levels in tumor tissue specimens with disease-specific survival (DSS) and overall survival (OS) in the subgroup of ccRCC patients with organ-confined tumours (tumour stage pT1+2, n = 87) assessed by multivariate Cox's regression analysis.

Factor	No. cases	Disease-specific survival HR (95% CI)[a]	*P*	Overall survival HR (95% CI)[a]	*P*
**Gender**					
male	47	1		1	
female	40	0.87 (0.29-2.59)	0.796	1.80 (0.78-4.15)	0.164
**Age (years)**					
≤ 64	48	1		1	
> 64	39	2.83 (0.89-8.92)	0.077	2.73 (1.23-6.10)	0.014
**Tumour grade**					
G1+2	58	1		1	
G3+4	29	1.56 (0.52-4.67)	0.428	1.34 (0.62-2.90)	0.456
**uPA**[b]					
low	51	1		1	
high	36	1.69 (0.54-5.32)	0.368	0.88 (0.39-1.97)	0.755
**uPAR**[b]					
low	49	1		1	
high	38	3.09 (0.88-10.8)	0.077	1.20 (0.52-2.73)	0.671
**PAI-1**[b]					
low	48	1		1	
high	39	1.59 (0.49-5.21)	0.441	1.33 (0.59-2.98)	0.486

[a]HR: hazard ratio; 95% CI: 95% confidence interval of multivariate Cox's regression analysis; uPA system factors were separately added to the base model consisting of gender, age and tumour grade.

[b]Dichotomised into groups with high and low levels of uPA system factors in tumour tissue by the median values.
